# Moderate systemic therapeutic hypothermia is insufficient to protect blood-spinal cord barrier in spinal cord injury

**DOI:** 10.3389/fneur.2022.1041099

**Published:** 2022-11-15

**Authors:** Rubing Zhou, Junzhao Li, Ruideng Wang, Zhengyang Chen, Fang Zhou

**Affiliations:** ^1^Department of Orthopedics, Peking University Third Hospital, Beijing, China; ^2^Neuroscience Research Institute and Department of Neurobiology, School of Basic Medical Sciences, Peking University Health Science Center, Beijing, China

**Keywords:** spinal cord injury, hypothermia, blood-spinal cord barrier, two-photon microscopy, tight junctions

## Abstract

Blood–spinal cord barrier (BSCB) disruption is a pivotal event in spinal cord injury (SCI) that aggravates secondary injury but has no specific treatment. Previous reports have shown that systemic therapeutic hypothermia (TH) can protect the blood–brain barrier after brain injury. To verify whether a similar effect exists on the BSCB after SCI, moderate systemic TH at 32°C was induced for 4 h on the mice with contusion-SCI. *In vivo* two-photon microscopy was utilized to dynamically monitor the BSCB leakage 1 h after SCI, combined with immunohistochemistry to detect BSCB leakage at 1 and 4 h after SCI. The BSCB leakage was not different between the normothermia (NT) and TH groups at both the *in vivo* and postmortem levels. The expression of endothelial tight junctions was not significantly different between the NT and TH groups 4 h after SCI, as detected by capillary western blotting. The structural damage of the BSCB was examined with immunofluorescence, but the occurrence of junctional gaps was not changed by TH 4 h after SCI. Our results have shown that moderate systemic TH induced for 4 h does not have a protective effect on the disrupted BSCB in early SCI. This treatment method has a low value and is not recommended for BSCB disruption therapy in early SCI.

## Introduction

Spinal cord injury (SCI) damages neural tissue and causes severe disability, leading to motor, sensory and autonomic dysfunction (1). After the primary mechanical impact, secondary injury exacerbates the degree of injury and extends the range of tissue damage in SCI (2, 3), represented as the continuous deterioration of neurological function (4). The spinal cord has a vital barrier structure similar to the brain, called the blood–spinal cord barrier (BSCB), to separate neural tissue from the peripheral blood flow by selective molecular substance exchange (5, 6). The permeability of the barrier function in the BSCB requires the formation of tight junctions (TJs), which seal the contacts between the endothelial cells and act as selective gates to control the paracellular diffusion of ions and solutes (7, 8). In addition, the BSCB maintains the immune isolation of neural tissue from the peripheral immune system, preventing pathogens and peripheral immune substances from entering the neural tissue (9, 10). BSCB disruption predictably emerges soon after the primary injury and is the main factor that induces secondary injury in tissue damage and neuroinflammation (11–13). However, no effective strategy has been developed to protect the BSCB or restore its disrupted barrier function after SCI (10).

Systemic therapeutic hypothermia (TH) has been employed as adjuvant therapy in traumatic brain injury and SCI for nearly a century by decreasing the core temperature through surface or endovascular cooling (14–17). TH has been reported to have beneficial effects on brain and spinal cord functions after injury and during neurosurgery, such as reducing tissue damage and better functional outcomes (18). The general opinion is that TH reduces the rate of biological reactions and processes, therefore targeting broad pathological processes in injury, including excitotoxicity, neuroinflammation, necrosis, and free radical production (1), but its neuroprotective mechanism remains unconfirmed. Some data have shown that TH can prevent the breakdown of the blood–brain barrier (19, 20), probably due to a membrane- or barrier-stabilizing effect to decrease barrier permeability and edema formation (21, 22). Multiple studies have shown that TH at 33°C for 4 h reduced the barrier leakage as demonstrated by various tracers and attenuated the local inflammatory response in brain trauma (23–26), while the underlying mechanism has not been fully verified. This effect was also suggested in human SCI patients, presenting as reduced tissue edema (27), along with improved motor neuron activity and blood supply in the spinal cord (28). However, these reports did not fully verify the underlying mechanism by which TH reduces barrier leakage.

Based on the current situation that no specific treatment is available to protect the BSCB after SCI, we aimed to test whether TH is sufficient to protect the BSCB or relieve BSCB leakage after SCI. We applied the typical procedure that has been proven to reduce blood–brain barrier leakage in animal brain trauma to the SCI model. *In vivo* two-photon microscopy was employed to monitor the BSCB leakage, combined with traditional histological examination. In addition, the effect of TH for 4 h on the expression and structural changes of TJ proteins after SCI was examined.

## Materials and methods

### Animals

All animal procedures were approved by the Animal Welfare Committee of Peking University Health Science Center (Protocol No. LA2019018). C57BL/6 mice were purchased from the Department of Laboratory Animal Science of Peking University Health Science Center. Male mice 8–10 weeks of age and 23 ± 1 g of weight were randomly allocated into different groups. Only male mice were chosen because SCI patients are mainly adult men. All mice had ad libitum access to food and water in a specific pathogen-free environment with a 12-h light/dark cycle.

### Surgical procedure

The mice were anesthetized with intraperitoneally injected pentobarbital (70 mg/kg, subsequently 35 mg/kg per hour). Mice were placed on a feedback-controlled hot plate with a rectal probe to monitor their core temperature (RWD Life Science, CHN) and maintain it to within 37.0 ± 0.5°C in the normothermia (NT) group and the control (Ctrl) group and within 32.0 ± 0.5°C in the moderate therapeutic hypothermia (TH) group. Their pulse, respiratory rate, and blood oxygen concentration were monitored. The shaved back surface of the mice was sprayed with alcohol for cooling and disinfection. A midline incision ~1.5 cm in length was then made in the back. The muscles covering the vertebra were carefully separated before a bilateral laminectomy was performed on the T12 vertebral column to expose the dorsal spinal cord. The dura mater was not removed. The mild contusion-SCI model was generated by a New York University Impactor (RWD Life Science) equipped with a 10 g hammer dropped from a height of 6 cm. Mice in the control group only received bilateral laminectomy but not SCI. A gelatin sponge soaked in saline was placed on the spinal cord to keep it moist and stop any bleeding.

### Two-photon *in vivo* microscopy

Animals were intravenously administered 5% (w/v in saline) 40 kDa TIRTC-dextran (Sigma, USA) 30 min before imaging (*n* = 6 mice each group). Kwik-Sil (World Precision Instruments, USA) and dental cement were carefully applied around the tip of a custom-made spine fixation apparatus and the rostral and caudal spines to build a chamber for imaging (29). The imaging window was built large enough to permit the tip of the impact hammer after the first imaging pre-SCI. Two-photon imaging was performed with a Leica TCS SP8/DIVE microscope equipped with a Mai Tai DeepSee pulsed laser (Spectra-Physics, USA). Imaging was performed under an HCX IR APO L25×/0.95 water immersion objective (Leica, GER). TRITC was excited at 1,050 nm with identical imaging conditions in all groups. Images of 512 × 512 pixel fields were acquired with 2 μm increments of z-stacks. The3D projections of vessel segmentations were reconstructed in LasX (Leica). The integrated density of the extravascular fluorescence was measured in ImageJ (NIH, USA). In brief, the image stacks were projected by the “sum intensity” at each time point. The spatial position of the vessels was calibrated by “rigid body” transformation to avoid small misalignments in the time-lapse image series. The integrated intensity in the extravascular area was measured by the same region of interest manually drawn outside the vessels and applied to each image in the image series.

### Immunohistochemistry

Anesthetized animals were transcardially perfused with 6 U/ml heparin in PBS and 4% paraformaldehyde (PFA) for 1 or 4 h after SCI (*n* = 5 mice each group). Three spinal cord segments (including the injured segment and the two adjacent segments) were harvested, postfixed in paraformaldehyde overnight, and dehydrated in gradient sucrose solutions. The fixed tissue blocks were sectioned at a thickness of 8 μm. Every tenth slice was attached to a glass slide. The sections were incubated in 3% hydrogen peroxide with methanol for 30 min, 10% blocking serum for 2 h, and then primary anti-IgG antibody (Bethyl, USA. Cat# A90-131A, 1:800) overnight at 4 °C. The primary antibody was detected with the HRP-conjugated secondary antibody and 3′-diaminobenzidine (DAB) substrate kit (Zsbio, CHN). Negative control slides were incubated in 10% serum without primary antibody. The sections were scanned with the NanoZoomer Digital Pathology system (Hamamatsu, JPN), and the optical density of IgG was analyzed in ImageJ (NIH).

### Capillary western blot

TJ proteins were measured by an automatic capillary western blot system (Simple Western, ProteinSimple, USA) according to the manufacturer's instructions. Tissues were harvested 4 h after SCI (*n* = 5 mice each group). The membrane components used to check claudin-5 and occludin were extracted by the Mem-PER Plus kit (Pierce, USA). ZO-1 expression was measured in whole lysed tissue homogenate for it undetectable in membrane or plasma components. The samples from each mouse were not pooled together and were instead detected individually. In brief, the samples were denatured at 95°C for 5 min and diluted to the appropriate concentration with master mix (ProteinSimple). Claudin and occludin always have two bands in a capillary western blot when denatured at 95°C, so the samples were denatured at 37°C for 30 min in the claudin and occludin groups according to the manufacturer's advice. Each sample and the commercialized reagents provided by the manufacturer were loaded into the assay well plate (ProteinSimple) and repeated twice. Vinculin (Cell Signaling, USA. Cat# 13901, 1:400) was used as a loading control for ZO-1 (Invitrogen, USA. Cat# 40-2200, 1:200), and ATPase (Abcam, UK. Cat# ab76020, 1:500) for claudin-5 (Abcam, Cat# ab131259, 1:100) and occludin (Abcam, Cat# ab167161, 1:100). ATPase was used as a loading control for claudin-5 and occludin, and vinculin was used for ZO-1. The target proteins and their reference proteins could not be detected in the same well by the machine, so they were added in adjoining wells according to the manufacturer's advice. The molecular weights of the proteins were confirmed by the manufacturer's antibody vocabulary. The chemiluminescence intensity of the proteins was automatically evaluated by Compass software (ProteinSimple) (30). Virtual images of the lanes were only used as representative images.

### Immunofluorescence

The tissue preparation was the same as for immunohistochemistry (*n* = 5 mice each group). The fixed tissue blocks were sectioned at a thickness of 35 μm. The sections were blocked with 2% PBST (2% Triton X-100 in PBS) containing 5% goat serum for 30 min at 37°C and then incubated with the primary antibody diluted in 0.4% PBST containing 1.5% goat serum for two nights at 4°C. The primary antibodies included claudin-5 (Bioworld, USA. Cat# BS1069, 1:200), occludin (Invitrogen, Cat# 40-4700, 1:150) and ZO-1 (Invitrogen, Cat# 40-2200, 1:150). The sections were washed with 0.4% PBST and incubated with secondary antibodies and DAPI (Abcam, Cat# ab150157, 1:500) diluted in 0.4% PBST containing 1.5% goat serum overnight at 4°C. The tissue sections were washed and mounted on glass slides. Negative control slides were incubated in 10% goat serum without primary antibody.

Images were taken with a Leica TCS STED microscope (Leica) equipped with a 40× HC PL APO CS2 oil-immersion lens (NA 1.3). Images of TJs on non-capillary vessels with diameters ranging from 10–30 μm were captured in 512 × 512 pixel fields as z-stacks with 0.5 μm increments. The TJ discontinuities with more than a 70% reduction in fluorescence intensity were defined as gaps. The gaps were manually measured and counted with LasX software (Leica software) in a double-blind manner.

### Statistics

All statistical analyses were performed in Prism 9 (GraphPad Software, USA). The sample sizes were determined based on previous experiments in our laboratory and representative literature that performed similar experiments. The power calculations were performed in SPSS (IBM, USA). Five animals were estimated to achieve 78.5–90.5% effect in this study. Multiple group comparisons were conducted by one-way ANOVA followed by a *post hoc Bonferroni analysis*. Time-lapse data were analyzed by two-way ANOVA followed by a *post hoc Bonferroni analysis*. No data were excluded from the statistical analysis. Details of the statistical results, including the exact *n* numbers, statistical tests, and definitions of significance, are presented in the figure legends. In all graphs, the data are expressed as the mean ± SEM; n.s. represents non-significant and ^*^P < 0.05, ^**^P < 0.01, ^***^P < 0.001, ^****^P < 0.0001.

## Results

### Therapeutic hypothermia does not reduce BSCB leakage after SCI

**Figure 1:**
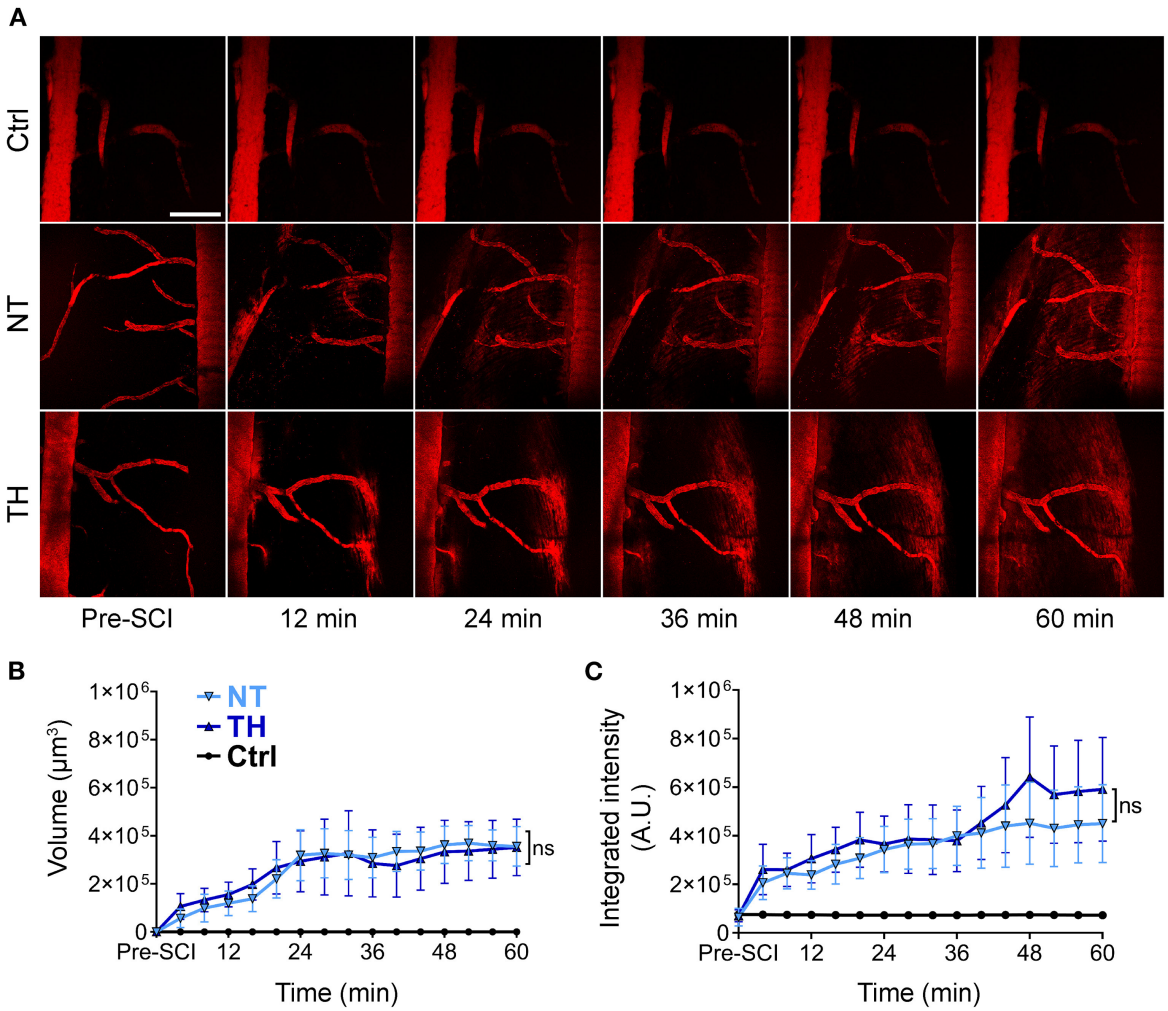
BSCB leakage of intravenously injected fluorescence-dextran in the injured segment after SCI. **(A)** Representative images of *in vivo* two-photon microscopy of the intravenously injected 40 kDa TRITC-dextran leaking from the disrupted BSCB an hour post-SCI. The 40 kDa TRITC-dextran did not leak from the normal BSCB pre-SCI. Scale bar: 200 μm. **(B)** The effect of TH on the volume of extravascular TRITC-dextran leaked from the disrupted BSCB an hour post-SCI (*P* = 0.997). **(C)** The effect of TH on the integrated intensity of the extravascular TRITC-dextran leaked from the disrupted BSCB with TH an hour post-SCI (*P* = 0.688). NT, normothermia; TH, therapeutic hypothermia; ns, non-significance. *n* = 6 mice per group, data are shown as mean ± SEM; nested, two-way ANOVA.

Intravascular tracers leaking into the neural tissue are the main indicator of barrier disruption. To monitor the process of BSCB leakage in real time, we used *in vivo* two-photon microscopy to trace the intravenously injected fluorescence-labeled dextran in the spinal cord segment pre-SCI and post-SCI (Figure 1A). The imaging endpoint was set to 1 h because the fluorescence intensity had a theoretical ceiling effect, and the outline of the blood vessels gradually became indistinct. Under physiological conditions, 40 kDa dextran does not freely pass through the barrier, while the fluorescence-labeled substances increased outside the vessels gradually after SCI, indicating that the BSCB leaked. Quantification of fluorescence-labeled indicators showed no difference between the NT and TH groups in either the volume (Figure 1B) or the integrated density (Figure 1C). These *in vivo* data show that applying TH cannot reduce BSBC leakage within 1 h after SCI.

**Figure 2:**
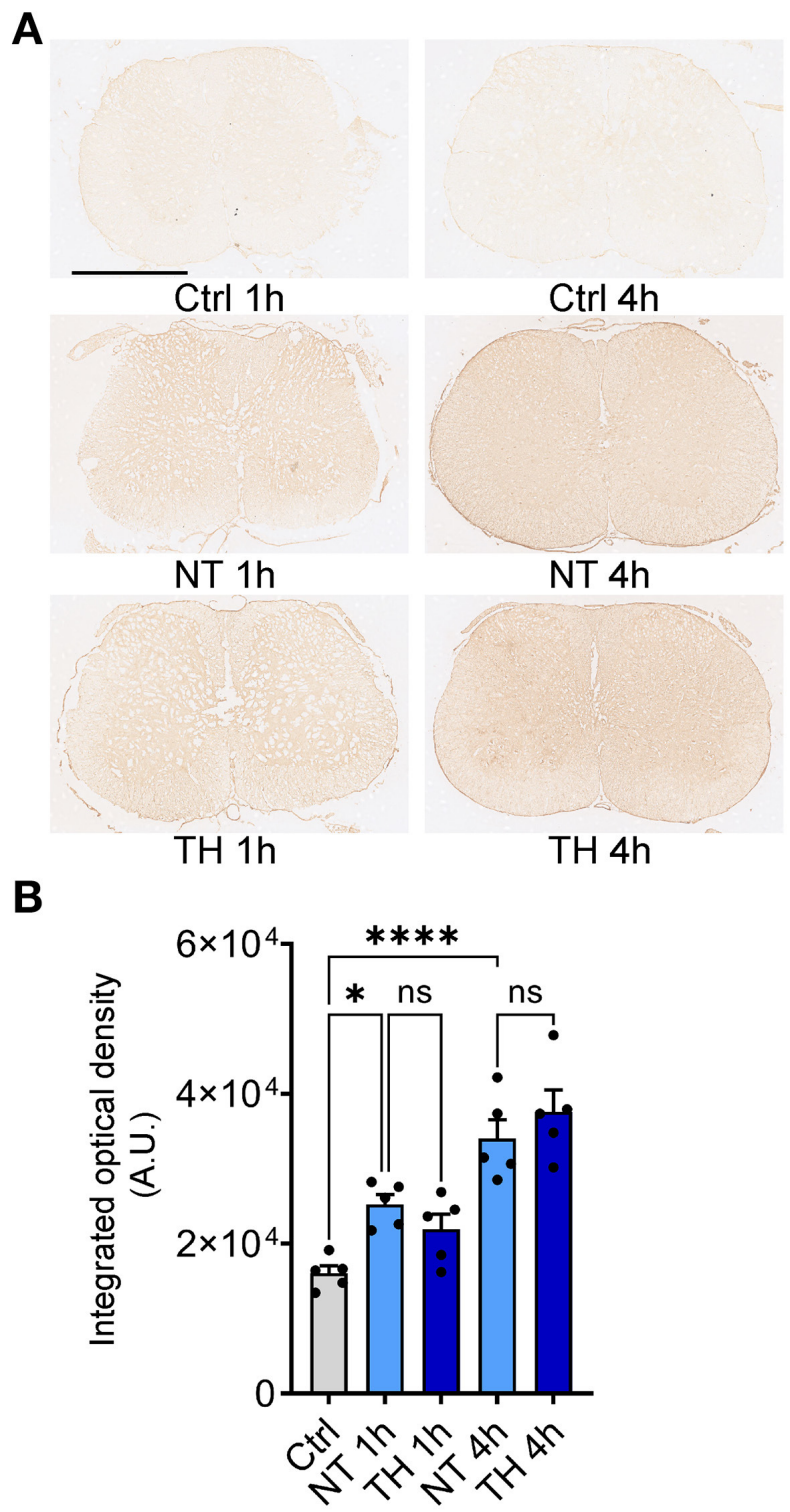
Serum non-specific immunoglobin G (IgG) leaked into the neural tissue after SCI. **(A)** Representative immunohistochemistry images of leaked serum IgG in the injured spinal cord (non-epicenter) 1 and 4 h post-SCI. Scale bar: 1 mm. **(B)** The integrated optical intensity of IgG in the impact and adjacent spinal segments with TH 1 and 4 h post-SCI. Normally, serum IgG does not exist in neural tissue. The optical density in the Ctrl group is the value of the tissue background. *n* = 5 mice per group, data are shown as the mean ± SEM; ns, non-significance; *P = 0.002, ****P < 0.0001, NT 1 h & TH 1 h: *P* = 0.716, NT 4 h & TH 4 h: *P* = 0.656; nested, one-way ANOVA with *Bonferroni's post hoc* test.

However, two-photon *in vivo* imaging cannot image the ventral side of the injured spinal cord due to the limited penetration of photons, so we employed the traditional histological method to determine the effect of TH on BCSB leakage after SCI. Peripheral immunoglobulin G (IgG) is not permitted to enter neural tissue under normal barrier function (31). Furthermore, the B lymphocytes generating IgG have been proven to migrate to the spinal cord several days after SCI (32). Hence, IgG in the spinal cord parenchyma in early SCI is thought to be present only if there is a disrupted barrier (33, 34). Immunohistochemical staining was performed on the injured and adjacent segments of the spinal cord 1 and 4 h after SCI (Figure 2A). The optical density of IgG in the spinal cord increased significantly after SCI, but applying TH resulted in no difference in IgG leakage compared to the NT group (Figure 2B). The *in vivo* and postmortem results suggest that TH cannot attenuate the leakage of different substances by disrupting the BSCB in the early period of SCI.

### Therapeutic hypothermia does not change the expression of tight junctions after SCI

**Figure 3:**
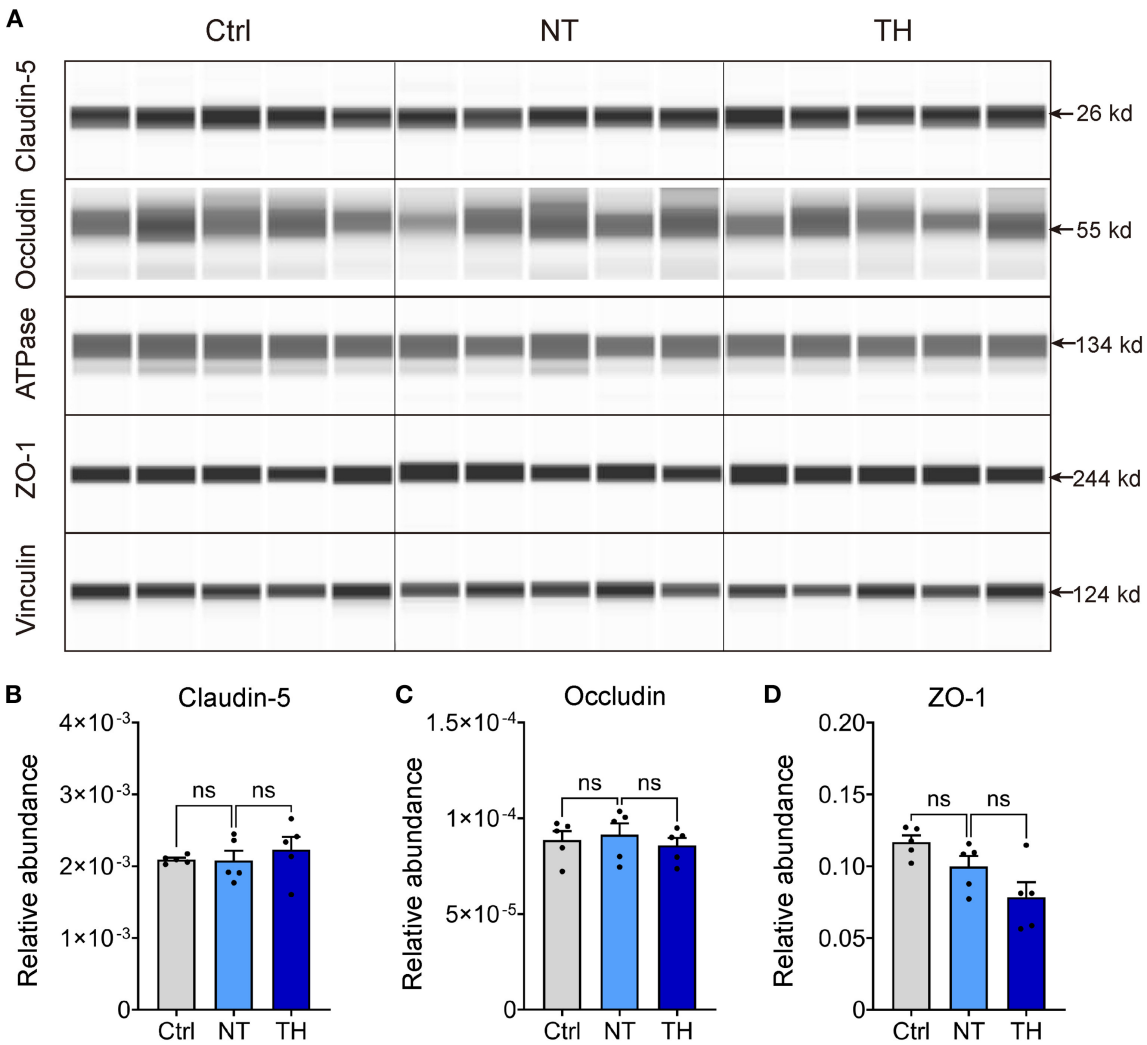
The expression change of endothelial tight junctions (TJ) with TH 4 h post-SCI. **(A)** Representative virtual lanes of capillary western blot analysis of TJ with TH 4 h post-SCI. These virtual lanes were generated from the ProteinSimple^TM^ system but not used for quantification. ATPase and vinculin were used as loading controls. **(B)** Quantification of the relative expression of claudin-5 with TH 4 h post-SCI (*n* = 5 mice per group, Ctrl & NT: *P* = 0.995, NT & TH: *P* = 0.856). **(C)** Quantification of the relative expression of occludin with TH 4 h post-SCI (*n* = 5 per group, Ctrl & NT: *P* = 0.99, NT & TH: *P* = 0.863). **(D)** Quantification of the relative expression of ZO-1 with TH 4 h post-SCI (*n* = 5 mice per group, Ctrl & NT: *P* = 0.28, NT & TH: *P* = 0.152). Data are shown as mean ± SEM; ns, non-significance; nested, one-way ANOVA with *Bonferroni's post hoc* test.

Previous studies showing that TH reduces the permeability of the blood–brain barrier did not reveal the exact underlying mechanism. Moreover, TH has some anticoagulatory effects (35–37), which could aggravate the microhemorrhage where the blood vessels are disrupted, along with the BSCB disruption presented by the leakage of intravascular tracers. Considering that these side effects can result in contradictory results, we next checked the effect of TH on the changes in TJs, which are the major foundation of the barrier in the endothelium (38, 39). The absolute content of TJs in neural tissue is relatively low for adequate detection by traditional western blotting without isolating microvessels from neural tissue (40–42). To overcome this problem, we performed capillary western blotting to detect TJ proteins, including claudin-5, occludin, and ZO-1 (Figure 3A). Compared to the non-injured spinal cord, the expression of claudin-5, occludin, and ZO-1 was not decreased 4 h after SCI. Meanwhile, therapeutic TH had no effect on the expression of TJs within 4 h after SCI (Figures 3B–D). SCI did not cause any significant expression changes in TJ proteins 4 h after SCI. Therefore, TH was not able to show a therapeutic effect on the expression of TJ proteins during this period.

### Therapeutic hypothermia does not alleviate the structural damage to tight junctions after SCI

**Figure 4:**
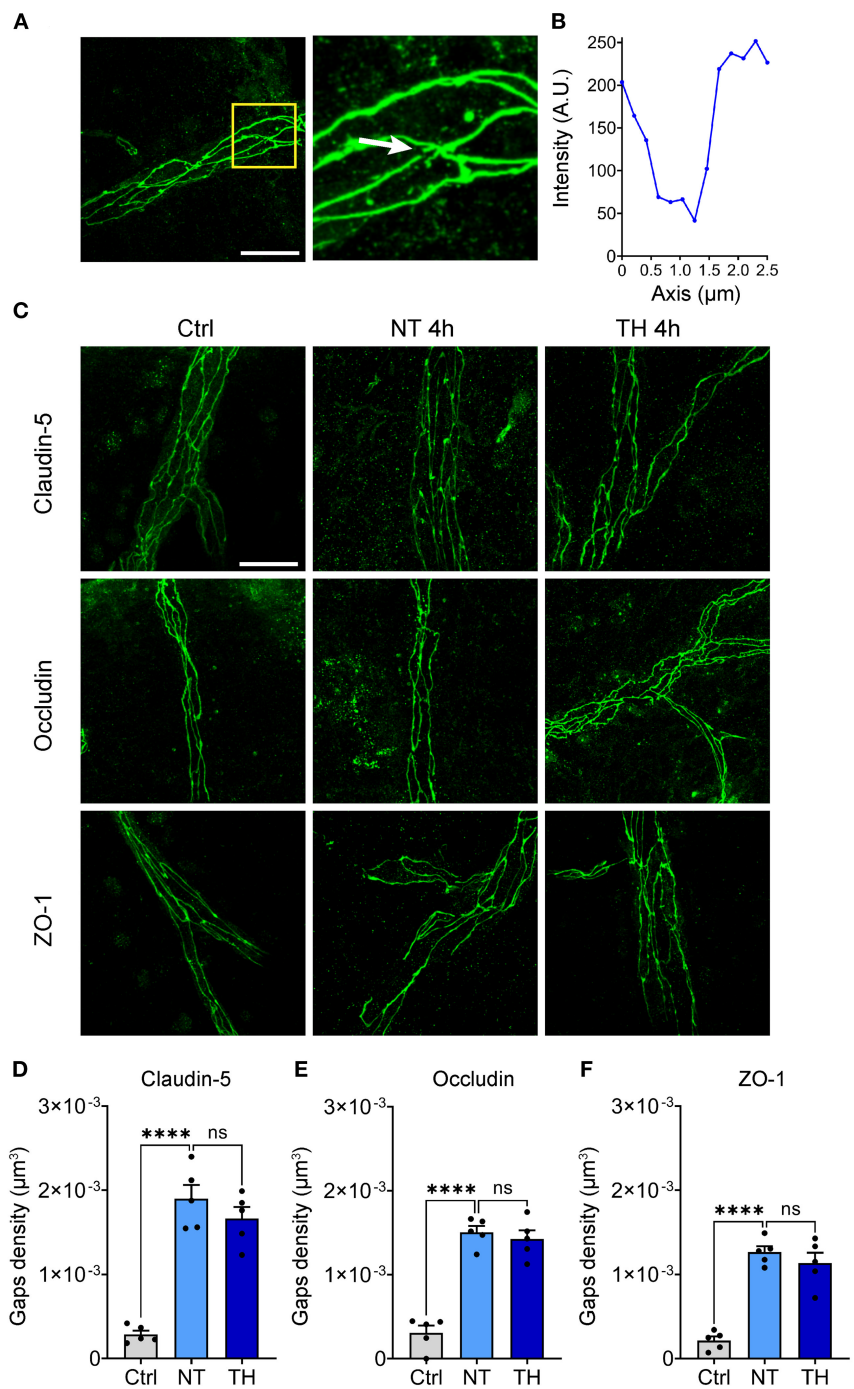
The effect of TH on structural damage to the TJs after SCI. **(A)** Representative images of the gaps in the TJs (white arrow). Scale bar: 20 μm. **(B)** Fluorescence intensity decline of the gap in Figure 1A. Fluorescence intensity declines of 70% were identified as a gap. **(C)** Representative images of immunofluorescence staining of TJs 4 h post-SCI. Scale bar: 20 μm. **(D)** The density of the gaps that emerged on claudin-5 with TH 4 h post-SCI (*n* = 5 per group, total 153 vessels, Ctrl & NT: *P* < 0.0001, NT & TH: *P* = 0.415). **(E)** The density of gaps that emerged on occludin with TH 4 h post-SCI (*n* = 5 per group, total 152 vessels, Ctrl & NT: *P* < 0.0001, NT & TH: *P* > 0.99). **(F)** The density of gaps that emerged on ZO-1 with TH 4 h post-SCI (*n* = 5 mice per group, total 157 vessels, Ctrl & NT: *P* < 0.0001, NT & TH: *P* = 0.609). Data are shown as mean ± SEM; ns, non-significance; ****P < 0.0001; nested, one-way ANOVA with *Bonferroni's post hoc* test.

Theoretically, the majority of BSCB leakage occurs in a paracellular manner by opening the paracellular junctions, while minor extravasation processes occur in a transcellular fashion transported within a vesicle-like structure through endothelial cells (43). BSCB leakage emerged, but the expression of TJs was not downregulated during this period, indicating that some other changes in TJs have occurred. To investigate the structural integrity of the TJs, immunofluorescence staining of claudin-5, occludin, and ZO-1 was performed 4 h after SCI. Many fluorescence discontinuities were observed among the TJs. We defined the fluorescence intensity decline by 70% as a TJ gap (Figures 4A,B). The TJ gaps increased significantly 4 h after SCI, but the density of TJ gaps was not different between the NT and TH groups (Figures 4C–F). These results indicate that TH cannot alleviate the structural damage to the BSCB in the early period after SCI.

## Discussion

BSCB disruption is a pivotal factor in the secondary injury process that further aggregates damage after SCI (44). Preventing BSCB disruption is a theoretically critical method for reducing SCI damage (45, 46), although no specific treatment has been proposed for the BSCB disruption (10). TH has been well established in treating nervous system injury (16, 17, 47) and has been reported to provide some effect in protecting the blood–brain barrier (19, 48) by reducing the barrier's permeability and alleviating edema (19, 49, 50). However, these observations did not elucidate the underlying mechanism of TH on the blood–brain barrier.

In our study, we used contusion SCI and combined *in vivo* and *in vitro* methods to check the effect of therapeutic TH on BSCB disruption during the early period of SCI, when BSCB leakage was most remarkable (51, 52). Two-photon microscopy and immunohistochemistry staining showed that applying TH for 1 and 4 h did not reduce BSCB leakage. Moreover, TH did not regulate the expression of TJs assessed by capillary western blotting and did not diminish the TJ gaps detected by immunofluorescence. Our data show that the application of 32°C TH for 4 h does not have sufficient potency to protect the BSCB in the early period after contusion SCI.

Previous studies on BSCB disruption were based on postmortem examinations, requiring harvesting the spinal cord from different animals at discontinuous time points, which requires sacrificing a large number of animals. To enable the dynamic monitoring of BSCB disruption, we utilized *in vivo* two-photon microscopy to record the leakage process in a time-lapse manner on individual mice before and after SCI, which is very suitable for tracing these dynamic processes (53). However, this *in vivo* approach has its drawbacks. Due to the limited penetration of photons, two-photon microscopy cannot image deeper tissue to view the ventral side of the spinal cord (54, 55). In addition, the massive vascular leakage blurs the boundaries of vessels over time. Therefore, we also employed the traditional immunohistochemistry method to monitor the BSCB leakage. The combination of the two methods can compensate for each other's shortcomings. Meanwhile, the experimental results were reconfirmed.

The mechanism of the neuroprotective effect of TH is still inconclusive (1). TH has a very complicated effect on multiple systems. Generally, TH probably reduces the rate of biological reactions and processes, thus decreasing many pathological processes after an injury (1). In clinical studies, clinical scores (such as the ASIA score) or functional tests (such as electrophysiology) are frequently used to detect the efficacy of TH, but without pathological study. These observation data cannot provide detailed clues to the mechanism of the observed neuroprotective phenomenon. Despite many successful reports showing improved motor and sensory function (16, 56), the neuroprotective effects of TH treatment for SCI are constantly inconsistent. The American Association of Neurological Surgeons (AANS/CNS) emphasizes its refusal to recommend TH for SCI until a multicenter randomized controlled trial is completed (57).

Previous data showed that 33°C TH for 4 h decreased the amounts of the different tracers entering the brain (23–26). However, a similar condition applied to the spinal cord did not show a positive result. One previous report found that profound TH (< 30°C) for 20 mins could slightly reduce plasma-derived albumin and fibrinogen-positive staining after compression SCI (58). However, the mechanism by which TH reduces BSCB leakage is unclear. Some researchers believe that TH can attenuate the permeability of the BSCB and reduce edema after SCI (59, 60) because TH could reduce the activity of multiple matrix metalloproteinases (MMPs) to relieve blood–brain barrier disruption or downregulate the water channel aquaporin-4 to reduce edema after brain injury (25, 61). Many reports have shown that MMPs increase after SCI (51), degrading TJ proteins and thus disrupting the BSCB (51, 62). In addition, some reports have shown that TH could reduce the level and activity of MMPs (24, 25). However, the MMPs start to increase significantly at ~8 h after SCI (63, 64), and it can even take days for key MMPs such as MMP-9 and MMP-12 to increase (63, 65), strongly suggesting that these enzymes do not play a pivotal role in early SCI. In this research, the level of TJ proteins was unchanged at 4 h after SCI, indicating that BSCB leakage in this period is not due to the degradation of TJ proteins, which MMPs usually cause. In addition, many gaps emerged in the TJs at 4 h after SCI. The structural changes in TJs reflect the barrier function of the BSCB (45, 51). The application of TH neither changed the expression of TJ proteins nor reduced the structural damage to the TJs, indicating that moderate TH does not have sufficient therapeutic efficacy to protect the BSCB early after SCI.

Moreover, systemic TH has a wide range of side effects on different systems. As observed in previous studies, a lower induced temperature of TH is accompanied by more severe complications (66). Profound TH (core temperature < 30°C) maintained for a long duration is not safe for mammals (21). In this study, we did not conduct experiments over a wider range of hypothermia. Reports from the University of Miami have shown that the optimized temperature for SCI patients is ~33°C (17), which is similar to our empirically selected temperature. Furthermore, a common complication of TH treatment is an anticoagulatory effect that worsens bleeding (17), including platelet, coagulation, and fibrinolysis dysfunction (21, 67). During TH treatment in experiments, physical injury-induced microhemorrhage could be amplified by coagulation disturbance and interfere with the quantitative analysis of BSCB disruption. Therefore, we only used mild contusion SCI to avoid bleeding. Additional measurements are needed to evaluate the role of TH in BSCB disruption after SCI to rule out the possibility that other factors may interfere with the effect of TH in reducing BSCB leakage.

Treating BSCB disruption after SCI requires fast-acting and effective treatments. The observations for 4 h in this study is relatively short and does not cover the entire acute period. However, TH can affect various systems and cause many complications. Prolonged TH is usually accompanied by more severe complications (66), which can be life-threatening and require further intensive care. Nevertheless, the duration of TH for human patients could be longer in the intensive care unit, while including long cooling induction and rewarming periods. Moreover, BSCB disruption is a key promotor of secondary injury, a self-promoted cascade that will peak at several hours after the injury. The protective effect of TH on the BSCB decreases over time because the BSCB disruption has already spread and aggravated secondary injury (2, 10, 68). Therefore, relieving the BSCB disruption at an early time point within the therapeutic window is essential. Assuming TH is effective for BSCB disruption, it needs to be applied within the early therapeutic window to have any clinical impact. In this study, TH was applied immediately after SCI, which provides the best opportunity to see a protective effect on the BSCB after SCI. However, the protective value of moderate TH treatment for the BSCB was insufficient.

In summary, this study explored the efficacy of TH in protecting the BSCB after SCI from multiple perspectives, including barrier leakage, the expression of TJs, and structural damage to the TJs. Based on the findings of this study, a 4-h TH may not have a therapeutic effect on BSCB disruption after SCI. The evidence base data drawn from brain injuries may not be appropriate for the spinal cord, probably due to the different organ structures or pathological mechanisms. Other methods to protect the BSCB or alleviate BSCB disruption after SCI require further exploration. In-depth research exploring the pathological mechanism of BSCB disruption is necessary to identify a more effective method for treating barrier disruption after SCI.

## Data availability statement

The raw data supporting the conclusions of this article will be made available by the authors, without undue reservation.

## Ethics statement

The animal study was reviewed and approved by Animal Welfare Committees of Peking University Health Science Center (Protocol No. LA2019018).

## Author contributions

RZ and FZ designed the experiments. RZ, JL, and RW performed the experiments. JL and ZC analyzed the data. RZ wrote the first draft. FZ reviewed and critiqued subsequent drafts. All authors contributed to the article and approved the submitted version.

## Funding

This work was mainly supported by the National Natural Science Foundation of China (81971160) and the Peking University Medicine Seed Fund for Interdisciplinary Research (BMU2018MX021).

## Conflict of interest

The authors declare that the research was conducted in the absence of any commercial or financial relationships that could be construed as a potential conflict of interest.

## Publisher's note

All claims expressed in this article are solely those of the authors and do not necessarily represent those of their affiliated organizations, or those of the publisher, the editors and the reviewers. Any product that may be evaluated in this article, or claim that may be made by its manufacturer, is not guaranteed or endorsed by the publisher.
